# Evaluation of exposure to volatile organic compounds (BTEX) and Polycyclic Aromatic Hydrocarbons (PAHs) in gas station workers and oxidative stress assessment in Karaj city

**DOI:** 10.1016/j.toxrep.2024.101767

**Published:** 2024-10-11

**Authors:** Asghar Ghahri, Pouria Seydi, Amir Ranjbar, Hosna Hatami, Tina Beheshti, Enayatollah Seydi

**Affiliations:** a Department of Occupational Health and Safety Engineering, School of Health, Alborz University of Medical Sciences, Karaj, Iran; b Research Center for Health, Safety and Environment, Alborz University of Medical Sciences, Karaj, Iran; c Department of Medical Surgical Nursing, School of Nursing and Midwifery, Mazandaran University of Medical Sciences, Iran; d Student Research Committee, Alborz University of Medical Sciences, Karaj, Iran

**Keywords:** Volatile organic compounds, Gas station, Polycyclic aromatic hydrocarbons, Occupational exposure, Oxidative stress

## Abstract

Gas stations are one of the sources of benzene, toluene, ethylbenzene and xylene (BTEX) and polyromantic hydrocarbons (PAHs). The present study was conducted with the aim of evaluating the level of breathing exposure of gas station workers to BTEX, PAHs and oxidative stress caused by exposure to these compounds in Karaj city. Oxidative stress and reactive oxygen species (ROS) is one of the mechanisms involved in the toxicity caused by exposure to gas vapors. In this study, all 25 gas stations in the city of Karaj were investigated. Personal sampling and analysis of BTEX and PAHs was done according to National Institute for Occupational Safety and Health (NIOSH) 1501 and 5515 methods, respectively. Finally, oxidative stress markers were investigated in 25 gas station workers and 25 control group. The results showed that the mean age and employment history of gas station workers are 39.96 ± 9.55 and 12.84 ± 6.92, respectively. Also, no significant difference was reported between gas station workers and control subjects in terms of oxidative stress parameters including the level of ROS, oxidized glutathione (GSSG) content, malondialdehyde (MDA) and reduced glutathione (GSH) content. The concentration values of personal exposure of gas station workers to BTEX and PAHs are lower than the occupational exposure limits (OEL). Although the level of oxidative stress parameters in gas station workers is higher than the control group, this difference is not statistically significant (p>0.05). It is recommended to take personal protection measures in case of chronic exposure.

## Highlights

• No significant difference was reported between gas station workers and control subjects in terms of oxidative stress parameters.
• The concentration values of personal exposure of gas station workers to BTEX is lower than the occupational exposure limits (OEL).
• The concentration values of personal exposure of gas station workers to PAHs is lower than the occupational exposure limits (OEL).
• It is recommended to take personal protection measures in case of chronic exposure.

## Graphical Abstract

**Figure:**
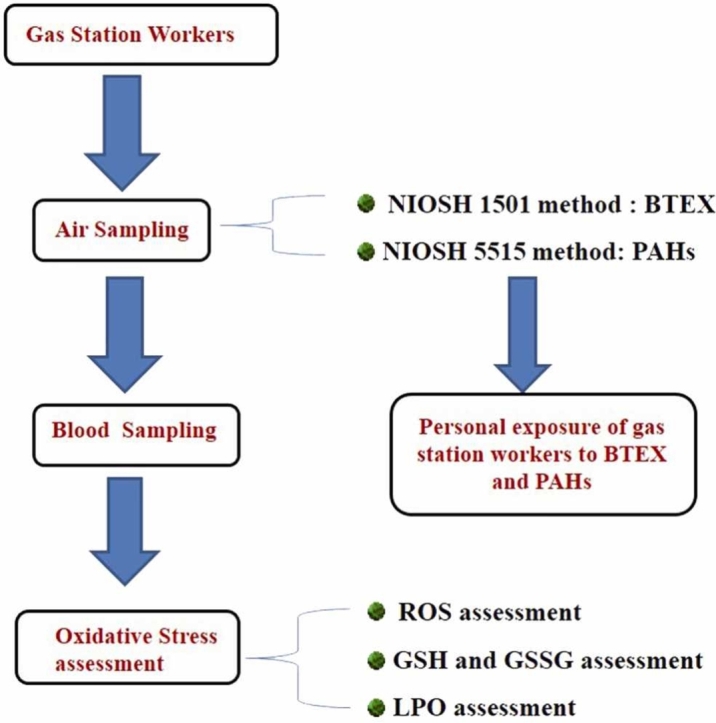

## 1 Introduction

Exposure to chemicals as dangerous pollutants in the workplace is one of the most important health problems. Various factors affect the risk created by these compounds [1], [2]. Overexposure to chemical pollutants in the workplace can affect the worker's health. Volatile organic compounds (VOCs) are known as one of the most important air pollutants and can lead to many complications. VOCs are widely used in oil-related industries and include thousands of different compounds [1], [2]. Petroleum products are xenobiotics that humans are exposed to benzene, toluene, ethylbenzene and xylene (BTEX), and polycyclic aromatic hydrocarbons (PAHs) are compounds derived from petroleum products. Gasoline/petrol is a clear liquid derived from petroleum, which consists of a mixture of hydrocarbon compounds. Car mechanics, drivers, and gas station employees are in direct exposure with gasoline vapors. Gas station workers are highly prone to occupational accidents and are exposed to physical, chemical, biological and physiological risk factors [3], [4], [5].

BTEX is one of the most important VOCs in the world, which is recognized as one of the most dangerous air pollutants. Several side effects such as carcinogenic and non-carcinogenic effects have been reported due to exposure to BTEX, which are dependent on the concentration and duration of exposure. Early and effective detection of BTEX is of great importance [6], [7]. In occupational settings, monitoring the level of exposure to these compounds is of great importance to identify their effects on workers and the environment [8]. Behbahanini *et al.,* study revealed that the concentration of benzene in all gasoline fuel distribution stations exceeded the environmental permissible limit [9]. In another study it was shown that all fuel stations had a lower BTEX value compared to the maximum exposure [10]. According to the study conducted by Mehrjerdi *et al.,* the mean concentration of benzene in a gas station in Yazd province is greater than the threshold limit value (TLV) [11]. It is also reported that gasoline stations had the highest concentration of benzene that exceeded the OEL-TWA [12].

In addition to BTEX exposure, gas station workers are also exposed to PAHs. PAHs are a group of compounds consisting of up to six benzene rings that result from pyrolysis or incomplete combustion of organic matter. Inhalation exposure is the most important route of exposure of workers to PAHs. These compounds have many toxic effects, including carcinogenic and mutagenic effects [13]. 16 PAHs have been prioritized as high-risk organic pollutants. Benzo[*a*]pyrene is a main PAHs that is considered as a cancer marker [14].

Petroleum products contain PAHs, and there is exposure with PAHs through inhalation of these products [15]. In occupational health, biological indicators are used to achieve special goals such as periodic personal monitoring of workers, exposure analysis of a group of workers, and epidemiological evaluation [16]. PAHs are mostly stored in the liver, lungs and kidneys. PAHs can be converted to simpler compounds or other metabolites and excreted through urine, feces and breast milk. Some of these metabolites are more toxic and harmful than their respective primary compounds. Due to the adverse effects of PAHs on humans and other living organisms, several studies have investigated the presence of these compounds in food, drinking water, air and other biological samples [17].

Petroleum vapor chemical pollutants, similar to other xenobiotics, may be metabolically converted to other metabolites that enter the body. After entering the body, these metabolites may become reactive and interact with the vital organs of the body to produce toxic effects. Interaction with these active compounds is associated with cell/tissue damage [4], [5], [18]. One of the main known mechanisms in toxicity caused by exposure to petrochemical products is oxidative stress and production of free radicals. These compounds have the ability to disrupt enzyme activities, and autoimmunity induction. Biotransformation of petroleum products with cytochrome-P450 (CYP2E1) is associated with an increase in the production of reactive oxygen species (ROS) [4], [5], [18].

Oxidative stress is a harmful process caused by an increase in the production of oxidant products [5]. There is a positive relationship between oxidative stress (production of ROS) and dysfunction of the body's vital macromolecules. These damages may be irreversible and are associated with changes in the function of different cells and cell death. The consequences of these irreversible damages are chronic diseases [19], [20]. PAHs can generate ROS and then cause oxidative damage to proteins, lipids, and deoxyribonucleic acid (DNA) [21]. Also, it has been shown that occupational exposure to PAHs causes oxidative stress in workers [22]. On the other hand, it has been shown that the exposure of gas station workers to BTEX is associated with a decrease in antioxidant activity in these workers [23]. Therefore, we assessed the level of exposure of gas station workers to PAHs and BTEX in Karaj city and the oxidative stress caused by exposure to these compounds.

## 2 Material and methods

### 2.1 Chemical

2,7-dicloroflurescein diacetate (DCHF-DA), O-pthaldialdehyde (OPA), *N*-ethylmaleimide (NEM), and ethylenediaminetetraacetic acid (EDTA) were purchased from Sigma Aldrich Company (St. Louis, MO, USA). In addition, all chemicals were of the highest commercial grade available.

### 2.2 Study design

The current research was a descriptive and cross-sectional study. Our research was conducted in Karaj city in 2023 and samples were collected from gas station workers. This study was approved by the ethics committee of Alborz University of Medical Sciences as ID: IR.ABZUMS.REC.1402.008. After became aware of our study all subjects (workers and controls) were asked to fill out the informed consent form. The study was performed in accordance with the ethical standards as laid down in the 1964 Declaration of Helsinki and its later amendments or comparable ethical standards.

### 2.3 Sampling, extraction and measurement

In this study, all 25 gas stations in the city of Karaj were selected. Sampling was done in winter. Also, the exposed samples were collected from gas station workers (25 male workers). Based on previous studies, 25 occupationally non-exposed persons were selected as the control group. The control workers was randomly selected from male employees of Alborz University of Medical Sciences. The inclusion criteria of the control workers included not exposed to the VOCs and PAHs compounds, non-chronic illness, non-smoking, non-alcohol consumption, and age between 20 and 65 years. NIOSH 1501 method was used to determine the level of personal exposure of gas station workers to BTEXs. In this method, a SOLID SORBENT TUBE was used for sampling. Also, gas chromatography (GC, Flame Ionization Detector (FID) detector/ Agilent 7890 A) was used for BTEX analysis. After sampling, the samples were immediately kept at a temperature below 4°C and transferred to the laboratory.

To extraction of BTEX, chemical desorption method and 1 ml of carbon disulfide (CS_2_ with purity of 99/5 %) were used. 30 s after adding CS_2_ to the vials, the samples were injected into GC. The injection temperature was 250 °C and the detector temperature was 300 °C. A capillary (fused silica) column was used for analysis. Helium (He) was used as carrier gas (flow rate: 2 ml/min). In the following, NIOSH 5515 method was used to determine the level of personal exposure of gas station workers to PAHs. In this method, a XAD-2 and PTFE filter (2-μm, 37-mm) was used for sampling. Also, gas chromatography (GC, FID detector) was used for PAHs analysis (PAH Standard Solution 6 components: Benzo (b) fluoranthene; Benzo(k)fluoranthene; Benzo(g,*h*,i)perylene; Benzo(a)pyrene; Indeno (1,2,3-c,d) pyrene; Fluoranthene). Acetonitrile and Ultrasonic bath were used to extraction of PAHs. The injection temperature was 200 °C and the detector temperature was 250 °C. Fused silica capillary column was used for analysis. He was used as carrier gas (flow rate: 1 ml/min).

Following the validation method of NIOSH 1501 (BTEX/ https://www.cdc.gov/niosh/docs/2003–154/pdfs/1501.pdf) and NIOSH 5515 (PAHs/ https://www.cdc.gov/niosh/docs/2003–154/pdfs/5515.pdf), the limit of detection (LOD) was carried out. The correlation coefficient (r) values was 0.99 for the chemicals. Also, control samples (blank) were tested in field sampling and laboratory analysis in order to check contamination levels and possible errors during sampling, transfer and analysis. The inclusion criteria included work experience of more than 5 years, absence of illness, non-smoking, non-alcohol consumption, 8-hour work shift, and age between 20 and 65 years.

### 2.4 Blood collection

10 ml of blood samples were obtained from gas station workers and the control group. Then, the collected blood was divided into two parts, and oxidative stress parameters such as ROS level were measured in blood (whole blood), and reduced glutathione (GSH) content, oxidized glutathione (GSSG) content and lipid peroxidation (LPO) level were measured in blood plasma.

### 2.5 ROS assay

At first, whole blood samples were collected from both groups (gas station workers and the control subjects), and then were suspended in the buffer for measuring ROS generation. To assess ROS, whole blood samples were incubated with Dichloro-dihydro-fluorescein diacetate (DCFH-DA) probe at concentration of 1.6 µM (30 min at 37 ° C). This probe is used to measure the ROS generation. The non-fluorescent DCFH and ROS reaction led to the formation of the highly fluorescent DCF. In the next step, fluorescence intensity (DCF) were assayed using a fluorescence spectrophotometer at the EXλ= 488 nm and EMλ= 527 nm [24]. Fluorescence intensity and ROS level have a direct correlation.

### 2.6 LPO assay

Malondialdehyde (MDA) is investigated as an indicator of LPO. MDA content in blood plasma samples from both groups (gas station workers and the control subjects) were assessed. In order to measure this parameter, MDA reacts with TBA and produces a colored thiobarbituric acid (TBA)-MDA complex. Then, this complex was measured at a wavelength of 532 nm using an ELISA reader. There is a positive relationship between the measured absorbance and the content of LPO [25].

### 2.7 Glutathione (GSH) and oxidized glutathione (GSSG) content assay

GSH and GSSG are important indicators for measuring the oxidative stress process. GSH and GSSG content in blood plasma samples from both groups (gas station workers and the control subjects) were assayed. The reaction of GSH with the *o*-phthalaldehyde (OPT) produces a compound that is highly fluorescent. Also, the reaction of GSSG with the OPT produces a fluorescent compound. Finally, Then, GSH and GSSG content was measured at the EXλ= 350 nm and EMλ= 420 nm using a fluorescence spectrophotometer. In oxidative stress, GSH content decreases and GSSG content increases [26].

### 2.8 Statistical analysis

The data were represented as Mean ± SD. The statistical tests were done using GraphPad Prism software (version 8). Independent t-test was used to analyze the data. Statistical significance was set at P<0.05. In this research, the confidence interval (CI) was 95 %.

## 3 Results

### 3.1 Average age and work experience

In this study, 25 gas stations in different central parts of Karaj city were studied. One person was randomly selected from each gas station as a sample. Our results indicated that the mean age of gas station workers is 39.96 ± 9.55 (95 % CI: 36.0179, 43.9021) and the mean work experience is 12.84 ± 6.92 (95 % CI: 9.98101, 15.699) (Table 1). Also, the results indicated that the mean age of the control group is 41.24 ± 8.01 (95 % CI: 37.9329, 44.5471) and the mean work experience is 11.52 ± 4.98 (95 % CI: 9.4626, 13.5774) (Table 1). According to the obtained results, no significant statistical difference was observed between the two groups.

**Table 1:** Mean age and work experience. Data are shown Mean ± SD (n=25).

**Groups**	**Age**	**Work experience**
**Control**	41.24 ± 8.01	11.52 ± 4.98
**Gas station workers**	39.96 ± 9.55	12.84±6.92

### 3.2 Concentration of BTEX and PAHs compounds

The measurement of the mean concentration of personal exposure of gas station workers to BTEX compounds in 25 locations showed that the mean concentration of exposure to benzene, toluene, ethylbenzene and xylene was 0.114 ± 0.143 ppm (95 % CI: 0.0112534, 0.216957), 0.085 ± 0.072 ppm (95 % CI: 0.0339287, 0.137303), 0.008 ± 0.006 ppm (95 % CI: 0.00412404, 0.0132468) and 0.031 ± 0.027 ppm (95 % CI: 0.0111417, 0.0511703), respectively (Table 2). Also, the concentration values of PAHs showed that the mentioned compounds were not detectable (Table 2). Based on occupational exposure limits (OEL), the time-weighted average (TWA) of exposure to benzene, toluene, ethylbenzene and xylene was 0.5 ppm, 20 ppm, 20 ppm, and 100 ppm, respectively. Our results of the study indicated that the level of personal exposure of gas station workers to BTEXs is lower than the recommended limit.

**Table 2:** Concentration values of personal exposure of gas station workers to BTEX and PAHs compounds. Data are shown Mean ± SD (n=25). ND: Not-detected.

**Compound**	**Concentration**	
	**ppm**	**mg/m**^**3**^
**Benzene**	0.114±0.143	0.364±0.459
**Toluene**	0.085±0.072	0.322±0.272
**Ethyl Benzene**	0.008±0.006	0.037±0.027
**Xylene**	0.031±0.027	0.135±0.121
**PAHs**	ND	ND

### 3.3 ROS level in gas station workers

**Fig. 1:**
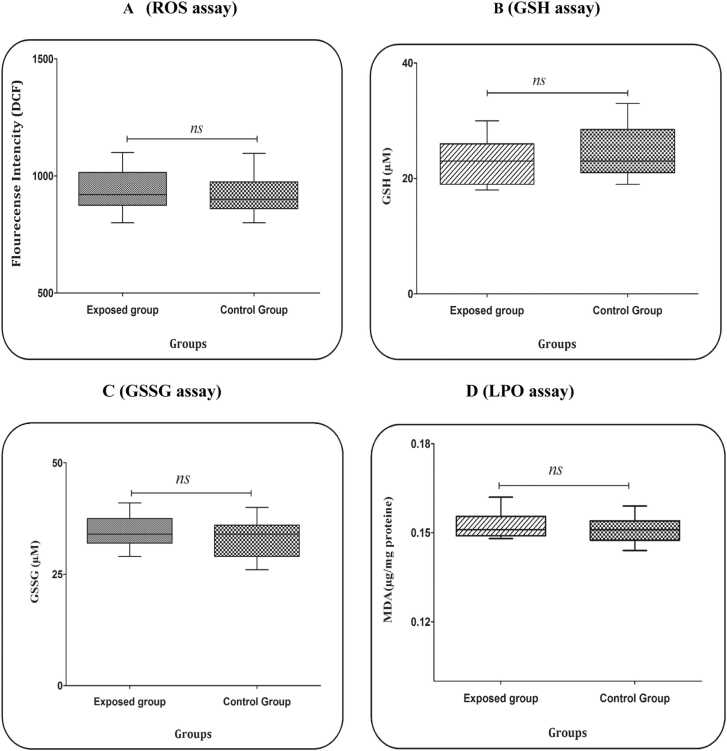
(A) ROS assay. Determination of ROS was measured through DCFH-DA probe in the blood sample (workers and controls). In this method, the fluorescence intensity indicates the ROS level. Independent t-test was used to analyze the data. Data are showed as mean ± SD (n = 25). Exposed group= gas workers. (B) GSH assay. The blood plasma samples (workers and controls) were used to measure GSH. Independent t-test was used to analyze the data. Data are showed as mean ± SD (n = 25). (C) GSSG assay. The blood plasma samples (workers and controls) were used to measure GSSG. Independent t-test was used to analyze the data. Data are showed as mean ± SD (n = 25). (D) LPO assay. MDA is investigated as an indicator of LPO. The blood plasma samples (workers and controls) were used to measure LPO. Independent t-test was used to analyze the data. Data are showed as mean ± SD (n = 25).

According to the obtained data, our report did not show a significant difference between the two groups in terms of ROS production (Fig. 1 A). Although the level of ROS was higher in gas station workers, this difference was not statistically significant (Fig. 1 A). The 95 % CI for the gas station workers was 903.048, 977.352. The 95 % CI for the gas station workers was 885.274, 946.006.

### 3.4 GSH content in gas station workers

As shown in Fig. 1B, there is no statistically significant difference between the content of GSH in gas station workers (95 % CI: 21.5155, 24.7245) and the control group (95 % CI: 22.9993, 26.4407). The average content of GSH in gas station workers is lower than the control group, but this difference is not statistically significant (Fig. 1B).

### 3.5 GSSG content in gas station workers

According to the obtained data, our report did not show a significant difference between the two groups in terms of GSSG content (Fig. 1 C). Although the GSSG content was higher in gas station workers, this difference was not statistically significant (Fig. 1 C). The 95 % CI for the gas station workers was 32.8523, 35.7877. The 95 % CI for the gas station workers was 31.459, 34.781.

### 3.6 LPO content in gas station workers

As shown in Fig. 1D, there is no statistically significant difference between LPO content in both groups. The average content of LPO in gas station workers (95 % CI: 0.150938, 0.154742) was higher than the control group (95 % CI: 0.149067, 0.152373), but this difference is not statistically significant (Fig. 1D).

## 4 Discussion

Research on the mechanisms that contribute to the association between occupational exposure of gas station workers with BTEX/PAHs and oxidative stress profiles are still limited. Our understanding of the adverse health risks caused by BTEX/PAHs exposure could be enhanced by exploring the molecular mechanism. Therefore, the aim of this research was to measure the occupational exposure to BTEX/PAHs in gas station workers in Karaj city, and analyze associations of BTEX/PAHs occupational exposure with oxidative stress profiles in gas station workers. In workplace, monitoring exposure to chemicals is necessary to establish a safe work environment [27]. Fuel distribution stations are one of the most important sources for the release of BTEX and PAHs, and the workers of these stations are in exposure with these dangerous substances. BTEX is one of the toxic xenobiotic that are found in the breathing zone of petrol station workers. BTEX is known to have a number of side effects on the body and is considered a dangerous compound based on research [28]. PAHs are dangerous xenobiotic derived from petrochemical products [29]. PAHs are compounds that are extremely harmful to living organisms due to their toxic, carcinogenic, resistant, and mutagenic properties [30]. Gas station workers are exposed to solvents and air pollutants in their workplace. BTEX exposure in the workplace has been identified as one of the most important occupational hazards [31].

The OEL based on the Iran Occupational Health Committee for BTEX are as follows, benzene 0.5 ppm (3.2 mg/m^3^), toluene 50 ppm (205.5 mg/m^3^), ethyl benzene 100 ppm (473 mg /m^3^), and xylene 100 ppm (473 mg/m^3^) [32]. Our findings revealed that the level of BTEX exposure in personal exposures obtained from workers at 25 gas stations is lower than the OEL. Furthermore, an examination of the gas station workers revealed that the concentration of exposure to PAHs was not detected. Allahabady *et al.,* results showed that the concentration of benzene in all gas stations is higher than the OEL, which is not in agreement with the result of our study. But the concentration of toluene, ethyl benzene and xylene is lower than the permissible limit, which is in agreement with our results. Differences in benzene concentration may be attributed to conditions including atmospheric conditions, location and fuel type [32]. According to Salama *et al*., study in Saudi Arabia, fuel stations have higher BTEX concentration values than limit for air quality standards values. Our study results are not in line with these results [33]. Similar to the study by Behbahanini *et al*., the results of our study also indicated that the concentration of PAHs compounds could not be detected after personal sampling in all gas stations under study [9].

The mechanism of oxidative stress is recognized as an important factor in chemical toxicity [34]. Oxidative stress is one of the research fields that many studies have focused on. This event is caused by the increase in the production of free radicals in tissues and cells and the ineffectiveness of defense systems against the removal of these by-products. Oxidative stress is a contributor to the pathogenesis of various diseases [35], [36], [37]. Oxidative stress is recognized as one of the crucial mechanisms that contribute to BTEX toxicity [38], and occupational studies have shown that exposure to BTEX is associated with oxidative stress. The mechanism by which BTEX causes oxidative stress is through the increase of ROS level and inhibition of antioxidant enzymes [7], [34], [39]. In addition, oxidative stress is known as the mechanisms involved in PAHs toxicity [40], [41]. GSH and superoxide dismutase are known as important antioxidant agent that protect cells during BTEX oxidation [7]. The study demonstrated that there were no significant differences in the levels of oxidative stress parameters (ROS, LPO, GSH and GSSG) between the two groups (control and gas station workers). Although, the levels of ROS, LPO, GSSG and GSH (as an important antioxidant agent) was higher in the control group than the gas station workers, but this difference is not significant. This no significance in the level of oxidant and antioxidant factors between the two groups may be due to the low level of exposure to BTEX and PAHs compounds. Elshaer *et al.,* showed that the exposure of petroleum workers to BTEX was associated with oxidative stress through the inhibition of antioxidant enzymes [39]. Uboh *et al.,* research demonstrated that BTEX/its metabolites are linked to gasoline-induced oxidative stress in rats [34]. The results of our study were not in agreement with these results. In our study, the workers were exposed, but in the study of Uboh *et al.,* the target group was rat, which were administered with gasoline [34]. Another report showed that the level of exposure to BTEX and oxidative stress was significantly higher in street children that working at busy intersections [7].

The data of this research was obtained from the target group (gas station workers) and is not of the type of simulated studies (for example, animal study and then generalizing the data to humans). Accordingly, these data can be used for epidemiological studies. This research is one of the few studies that measure both the exposure level and important oxidative stress parameters in this target group. In our study, four important parameters of oxidative stress were investigated, which is different from other studies. The release of VOC can depend on weather conditions. Unfortunately, the measurement of these compounds during different seasons was not done.

It is recommended that urinary monitoring of biomarkers of BTEX and PAHs, and also measurement of these compounds in the air of gas stations using newer extraction methods, should be investigated in future studies. In addition, the authors suggest that the evaluation of the carcinogenicity of these compounds in gas station workers should be investigated. Future studies require the examination of respiratory status, the use of antioxidant agents, and continual monitoring of control methods.

## 5 Conclusion

According the results of this study the mean concentration of BTEX and PAHs in all gas station worker was lower than the OEL. Furthermore, there was no significant differences in the levels of oxidative stress between the gas station workers and controls. However, oxidative stress is a process that can have different side effects on worker's health in the long time. Therefore, it is crucial to emphasize the use of personal protective equipment and control methods.

## CRediT authorship contribution statement

**Pouria Seydi:** Writing – original draft, Data curation. **Asghar Ghahri:** Writing – original draft, Software, Methodology, Investigation, Formal analysis. **Enayatollah seydi:** Writing – original draft, Supervision, Software, Project administration, Methodology, Investigation, Data curation, Conceptualization. **Tina Beheshti:** Writing – review & editing, Methodology. **Hosna Hatami:** Writing – original draft, Data curation. **Amir Ranjbar:** Writing – original draft, Methodology, Data curation.

## Declaration of Competing Interest

The authors declare that they have no known competing financial interests or personal relationships that could have appeared to influence the work reported in this paper: Enayatollah Seydi reports was provided by Alborz University of Medical Sciences. Enayatollah Seydi reports financial support was provided by Alborz University of Medical Sciences. Enayatollah Seydi reports financial support was provided by Alborz University of medical Sciences. Enayatollah Seydi reports a relationship with Alborz University of Medical Sciences that includes: funding grants. Enayatollah Seydi has patent pending to no. If there are other authors, they declare that they have no known competing financial interests or personal relationships that could have appeared to influence the work reported in this paper.

## Data Availability

Data will be made available on request.

## References

[1] Golbabaie F. (2012). Health risk assessment of chemical pollutants in a petrochemical complex. Iran. Occup. Health.

[2] Harati B. (2017). Risk assessment of chemical pollutants in an automobile manufacturing. J. Health Saf. Work.

[3] Abubakar M.B. (2020). The effects of honey administration on petrol-induced oxidative stress and hepatotoxicity in sprague-dawley male rats. Ann. Clin. Exp. Med..

[4] Owagboriaye F. (2018). Assessment of the effect of gasoline fume on stress hormones, antioxidant status and lipid peroxidation in albino rat. J. king Saud. Univ. -Sci..

[5] Owumi S.E. (2021). Combine effect of exposure to petrol, kerosene and diesel fumes: On hepatic oxidative stress and haematological function in rats. Toxicol. Ind. Health.

[6] Choudhury S.P. (2020). BN quantum dots decorated ZnO nanoplates sensor for enhanced detection of BTEX gases. J. Alloy. Compd..

[7] Rafiee A. (2022). Exploring urinary biomarkers to assess oxidative DNA damage resulting from BTEX exposure in street children. Environ. Res..

[8] Mihajlović V. (2021). Assessment of Occupational Exposure to BTEX in a Petrochemical Plant via Urinary Biomarkers. Sustainability.

[9] Behbahaninia A., Motahari S. (2022). Evaluation of Concentration of BTEX and PAHs and Its Effective Parameters in Gasoline Fuel Distribution Stations: A Case Study in Single Purpose Stations in Karaj. J. Environ. Health Engine.

[10] Hasanpour A., Aminshrei F. (2021). Health risk assessment of BTEX concentration of Gasoline stations with vapor collection system on Workers in Isfahan. J. Environ. Sci. Stud..

[11] Mosaddegh Mehrjerdi M.H. (2014). The investigation of exposure to benzene, toluene, ethylbenzene and xylene (BTEX) with Solid Phase Microextr action Method in gas station in Yazd province. Iran. South Med. J..

[12] Javadi I. (2017). Occupational exposure of shahindej county refueling stations workers to BTEX compounds, in 2016. J. Res. Environ. Health.

[13] Moubarz G. (2022). Lung cancer risk in workers occupationally exposed to polycyclic aromatic hydrocarbons with emphasis on the role of DNA repair gene. Int. Arch. Occup. Environ. Health.

[14] Jalili V., Barkhordari A., Ghiasvand A. (2020). Liquid-phase microextraction of polycyclic aromatic hydrocarbons: A review. Rev. Anal. Chem..

[15] Rashid A. (2017). Petrol filling workers as biomonitor of PAH exposure and functional health capacity in resource-limited settings of city Rawalpindi, Pakistan. Environ. Sci. Pollut. Res..

[16] Verdonck J. (2021). Systematic review of biomonitoring data on occupational exposure to hexavalent chromium. Int. J. Hyg. Environ. Health.

[17] Gheshlaghi M., Farhadi K., Tahmasebi R. (2023). Gas Chromatographic Detection of Polycyclic Aromatic Hydrocarbons (PAHs) in Indoor Air After Direct Extraction by a Novel Monolithic Adsorbent. Anal. Bioanal. Chem. Res..

[18] Uhegbu F.O. (2017). Renal Protective Properties of Aqueous Extract of Bryophyllum pinnatum (Lam.) Oken Leaf against Petrol Vapour–Induced Toxicity on Male Albino Rats. Eur. J. Med Plants.

[19] Andrés Juan, C., et al., The Chemistry of Reactive Oxygen Species (ROS) Revisited: Outlining Their Role in Biological Macromolecules (DNA, Lipids and Proteins) and Induced Pathologies. 2021. 10.3390/ijms22094642 PMC8125527 33924958

[20] zargari f (2020). The role of oxidative stress and free radicals in diseases. Razi J. Med. Sci..

[21] Xiao Q. (2022). Exposure to polycyclic aromatic hydrocarbons and the associations with oxidative stress in waste incineration plant workers from South China. Chemosphere.

[22] Zeng H. (2022). Combined effects of exposure to polycyclic aromatic hydrocarbons and metals on oxidative stress among healthy adults in Caofeidian, China. Ecotoxicol. Environ. Saf..

[23] Rizk A.A. (2020). Assessment of oxidative stress among refueling workers in an Egyptian setting. Environ. Sci. Pollut. Res..

[24] Ghahri A. (2021). The polycyclic aromatic hydrocarbons (PAHs)-induced toxicity in asphalt workers neutrophils through induction of oxidative stress. Toxicol. Environ. Health Sci..

[25] Beach D.C., Giroux E. (1992). Inhibition of lipid peroxidation promoted by iron (III) and ascorbate. Arch. Biochem. Biophys..

[26] Hissin P.J., Hilf R. (1976). A fluorometric method for determination of oxidized and reduced glutathione in tissues. Anal. Biochem..

[27] Moro A.M. (2013). Genotoxicity and oxidative stress in gasoline station attendants. Mutat. Res. /Genet. Toxicol. Environ. Mutagen..

[28] Alimohammadi M. (2023). Carcinogenic and Health Risk Assessment of Respiratory Exposure to BTEX Compounds in Gasoline Refueling Stations in Karaj–Iran.. Pollution.

[29] Lin X.-Y. (2022). Polycyclic aromatic hydrocarbon exposure and DNA oxidative damage of workers in workshops of a petrochemical group. Chemosphere.

[30] Thang P.Q. (2019). Seasonal characteristics of particulate polycyclic aromatic hydrocarbons (PAHs) in a petrochemical and oil refinery industrial area on the west coast of South Korea. Atmos. Environ..

[31] Al-Harbi M. (2020). Health symptoms associated with occupational exposure of gasoline station workers to BTEX compounds. Atmos. Environ..

[32] Allahabady A. (2022). Measurement of BTEX (benzene, toluene, ethylbenzene and xylene) concentration at gas stations. Environ. Health Eng. Manag. J..

[33] Salama K.F., Omer E.O., Zafar M. (2021). Assessment of BTEX concentration around fuel station in Eastern Province Kingdom of Saudi Arabia. Int. J. Environ. Health Eng..

[34] Uboh F.E. (2022). Benzene, toluene, ethylene and xylene (BTEX) is implicated in gasoline-induced oxidative stress in male albino Wistar rats. J. Toxicol. Environ. Health Sci..

[35] Aranda-Rivera A.K. (2022). RONS and oxidative stress: An overview of basic concepts. Oxygen.

[36] Olufunmilayo E.O., Gerke-Duncan M.B., Holsinger R.D. (2023). Oxidative stress and antioxidants in neurodegenerative disorders. Antioxidants.

[37] Rotariu D. (2022). Oxidative stress–Complex pathological issues concerning the hallmark of cardiovascular and metabolic disorders.. Biomed. Pharmacother..

[38] Guo Y. (2023). Benchmark dose estimation based on oxidative damage in Chinese workers exposed to benzene series compounds. Environ. Toxicol. Pharmacol..

[39] Elshaer N. (2022). Evaluation of Oxidative Stress among Petroleum Workers Exposed to Benzene, Toluene, Ethylbenzene, and Xylene in Alexandria, Egypt. Evaluation.

[40] Zhang Y.-J. (2021).

[41] Zhu H., Martinez-Moral M.-P., Kannan K. (2021). Variability in urinary biomarkers of human exposure to polycyclic aromatic hydrocarbons and its association with oxidative stress. Environ. Int..

