# Supplementary material for: Evaluation of exposure to volatile organic compounds (BTEX) and Polycyclic Aromatic Hydrocarbons (PAHs) in gas station workers and oxidative stress assessment in Karaj city
Source: Toxicol Rep. 2024 Oct 11;13:101767. doi: 10.1016/j.toxrep.2024.101767 (PMC11525218; doi:10.1016/j.toxrep.2024.101767)
Supplement: Supplementary file 1 — Supplementary material [file mmc1.docx]

**The methods of NIOSH 1501 and 5515 have been explained precisely and step by step.**

**BTEX Determination:**

**NIOSH 1501 method** was used to determine the level of personal exposure of gas station workers to BTEXs.

NIOSH 1501 method was used to determine the level of personal exposure of gas station workers to BTEXs. In this method, a SOLID SORBENT TUBE was used for sampling. Also, gas chromatography (GC, Flame Ionization Detector (FID) detector/ Agilent 7890A) was used for BTEX analysis. To extrication of BTEX, chemical recovery method and 1 ml of carbon disulfide (CS_2_ with purity of 99/5%) were used. 30 s after adding CS_2_ to the vials, the samples were injected into GC. The injection temperature was 250 °C and the detector temperature was 300 °C. A capillary (fused silica) column was used for analysis. Helium (H_2_) was used as carrier gas (flow rate: 2 ml/min).

- SOLID SORBENT TUBE (coconut shell charcoal) and SKC sampling pump with a flow rate of 0.1 to 1 L/min were used for sampling.
- The mentioned tubes were placed inside the pump.
- After sampling, the samplers were kept at a temperature below 4°C and transported to the laboratory.
- After sampling, the adsorbent was placed in foil and transported to the laboratory by an ice flask.
- The duration of sampling is 1 hour at a height of 1 to 1.5 m from the floor (approximately the breathing range of worker).
- Activated charcoal in both the back and front parts of the absorbent tubes containing the sample were transferred to separate vials.
- To extract BTEX, chemical recycling method and 1 ml of carbon disulfide (CS_2_) solution with 99/5% purity were used.
- After adding carbon disulfide to the vials, at least 30 seconds of time was considered for complete extraction of BTEX compounds.
- After preparation, the analyte was quantified by gas chromatography model Agilent 7890A.
- In order to determine the amount of the desired analyte, 1 µL of the prepared solution should be injected by a 10 µL syringe made by Hamilton Company in the injection site of the device.
- Helium (He) gas was also used as a carrier gas with a flow rate of 2 ml/min.
- The information related to each chromatogram, including the height and area under each curve (AUC), was extracted.
- Finally, control samples (blank) were tested in field sampling and laboratory analysis in order to check contamination levels and possible errors during sampling, transfer and analysis.

**PAH determination:**

**NIOSH 5515 method** was used to determine the level of personal exposure of gas station workers to PAHs. In this method, a XAD-2 and PTFE filter (37 mm) was used for sampling. Also, gas chromatography (GC, FID detector) was used for PAHs analysis. Acetonitrile and Ultrasonic bath were used to extraction of PAHs. The injection temperature was 200 °C and the detector temperature was 250 °C. Fused silica capillary column was used for analysis. Helium (H_2_) was used as carrier gas (flow rate: 1 ml/min).

- Calibrate each personal sampling pump with a representative sampler in line.
- Take personal samples at 2 L/min for a total sample size of 200 to 1000 L.
- Immediately after sampling, transfer the filter carefully with forceps to a scintillation vial. Hold filter at edge to avoid disturbing the deposit. Cap the scintillation vial and wrap it in aluminum foil.
- PAHs were placed in the foil after sampling by the adsorbent.
- Then the samples were transferred to the laboratory in an ice flask.
- Acetonitrile and Ultrasonic bath were used to extraction of PAHs.
- The samples were placed in an ultrasonic bath for 30 minutes.
- In order to determine the amount of the PAHs, the prepared solution should be injected by a 10 µL syringe made by Hamilton Company in the injection site of the device.
- The information related to each chromatogram, including the height and area under each curve (AUC), was extracted.
- The injection temperature was 200 °C and the detector temperature was 250 °C.
- Fused silica capillary column was used for analysis.
- Helium (H_2_) was used as carrier gas (flow rate: 1 ml/min).
